# ﻿*Phloeosinusmetasequoiae* sp. nov. (Coleoptera, Curculionidae, Scolytinae, Phloeosinini), a new insect pest of *Metasequoiaglyptostroboides* in China

**DOI:** 10.3897/zookeys.1228.138084

**Published:** 2025-02-20

**Authors:** Hang Ning, Ruixiong Deng, Beibei Huang, Kaitong Xiao, Jingjing Huang, Jianfeng Hong, Yongmei Yi

**Affiliations:** 1 Hubei Key Laboratory of Biological Resources Protection and Utilization (Hubei Minzu University), Enshi 445000, Hubei, China; 2 College of Forestry and Horticulture, Hubei Minzu University, Enshi 445000, Hubei, China; 3 Xingdoushan National Nature Reserve, Enshi 445000, Hubei, China

**Keywords:** Bark beetle, *Metasequoiaglyptostroboides*, morphology, molecular, new species, taxonomy

## Abstract

We describe herein a previously unknown species of bark beetle, *Phloeosinusmetasequoiae* Ning, **sp. nov.**, which was discovered in the Xingdoushan National Nature Reserve, Hubei Province, China. This new species can be distinguished from other species in the genus *Phloeosinus* Chapuis, 1869 by its deeply emarginate compound eyes, coarse frontal and prothoracic surfaces, reticulate texture, and deeply V-shaped basal margin of the elytra. Phylogenetic analyses based on the cytochrome oxidase I (COI) and the large nuclear ribosomal subunit (28S) genes indicated that the new species represents an independent lineage with the closely related *Phloeosinusaubei* (Perris, 1885), to which it has a 95% similarity. The new species is known only from the type locality. Diagnoses, descriptions, photographs, and a distribution map are presented, along with a description of host plant damage.

## ﻿Introduction

*Metasequoiaglyptostroboides* Hu & W.C.Cheng (Cupressaceae) is a typical relictual tree species with a native distribution limited to an extremely narrow triangular area encompassing Lichuan City, Hubei Province, and Shizhu County, Chongqing Municipality, and Longshan County, Hunan Province (Ma and Shao 2003; Wang et al. 2004). This deciduous conifera is an endangered species and the sole living representative of the genus *Metasequoia* Hu & W.C.Cheng, which was once believed to have become extinct during the Miocene epoch (Equiza et al. 2006). Currently, there are an estimated 5779 native mother trees of *M.glyptostroboides*, among which, 5746, 3, and 28 are distributed in Lichuan City, Longshan County, and Shizhu County, respectively (Wang et al. 2005). The specimens in Lichuan City grow in Jiannan, Moudao, Wangying, and Zhonglu towns and the Fubao Mountain Forest Management Area (Liu et al. 2023). As an important relictual plant species, *M.glyptostroboides* has considerable ornamental, medicinal, and ecological value. The tree is characterized by an attractive pattern of growth and bears lush green leaves, and although the native population is now restricted to a very small area, it is widely cultivated for use as street and ornamental trees in parks and gardens. In China, the cultivation range of *M.glyptostroboides* extends across 26 provinces, with an estimated planting area of 1.08 × 10^4^ hm (Leng et al. 2007). *Metasequoiaglyptostroboides* is commonly found growing in wetland plains in the middle and lower reaches of the Yangtze River and coastal areas, and it has also been introduced to more than 50 countries worldwide, with a distribution range extending northward to 60°N in regions such as Volgograd and Alaska.

To date, comparatively few pests or diseases of the trunk have been reported for *M.glyptostroboides*, among which *Odontotermesformosanus* (Shiraki, 1909) is the most significant pest, and is considered to pose a threat to the survival of native mother trees (Wang et al. 2003). Given their weakened growth, mother trees that are characterized by hollow heartwood can be particularly vulnerable to infection by *O.formosanus*. Studies on pests that are detrimental impact to the growth of *M.glyptostroboides* have generally focused on defoliating insects, including *Ascotisselenaria* (Hubner, 1817), *Choristoneurametasequoiacola* (Liu, 1983), and *Ectropisobliqua* (Prout, 1915), whereas with respect to diseases, red blight, leaf blight, and leaf spot have been reported to be relatively common in *M.glyptostroboides* (Yang et al. 2021; Liang et al. 2023). However, previous studies tended to indicate that these pests and diseases are non-lethal and cause relative minor damage to the native mother trees. To date, however, there have been no reports of bark beetles feeding on *M.glyptostroboides*.

However, a recent survey of the pests of *M.glyptostroboides* in May to June 2024 in the Xingdoushan National Nature Reserve, Hubei Province, yielded what appeared to be a new species of bark beetle, which characterize here based on morphological observations and molecular analyses. Comparative morphological and molecular phylogenetic analyses provide evidence to indicate that the collected specimens represent a previously undescribed species, which we describe herein.

## ﻿Materials and methods

### ﻿Taxonomy

Specimens of the collected beetles were examined using a hand-held digital microscope (Aomekie A5; Aomekie, Ningbo City, China) and photographed using a DSLR camera (Canon EOS R6; Canon, Ota City, Kyoto, Japan) mounted on an Olympus BX53 compound microscope with ×5, ×10, and ×20 fluorite objectives (Olympus, Shinjuku City, Tokyo, Japan). Images were also obtained using a Canon EOS R6 camera equipped with a Godox Speedlite flash unit to provide additional lighting, with montage images being assembled using Adobe Photoshop 2023 (Adobe Inc., San Jose, California, USA). All measurements were made using a stereomicroscope (AxioVision SE64 v. 4.8.3) and are presented in millimeters. The descriptions and morphological terms used in the text are based on those adopted by Alonso-Zarazaga et al. (2023), Knížek (2011), Beaver (2011), Wood and Bright (1987, 1992), and Tsai and Yin (1964). Depositories of all specimens examined are abbreviated as:

**ASM-HBMZU** Animal Specimens Museum, College of Forestry and Horticulture, Hubei Minzu University, Enshi, Hubei, China.

### ﻿Abbreviations and terminology

**NZMC** National Zoological Museum of China, Institute of Zoology, Chinese Academy of Science, Beijing, China

**NMNS** National Museum of Natural Science, Taichung, Taiwan, China

**NMNH** National Museum of Natural History, Washington, DC, USA

**NHMW** Naturhistorisches Museum Wien, Austria

**NHMUK** The Natural History Museum, London, UK

### ﻿DNA extraction, PCR amplification, and sequencing

Live adult beetles were collected directly from beneath the bark of recently infested trees and preserved in 90% ethanol or stored at −80 °C until used for analyses. These specimens have been saved as vouchers in the Forest Conservation and Utilization Professional Laboratory, Hubei Key Laboratory of Biological Resources Protection and Utilization (Hubei Minzu University). DNA was extracted from specimens using a magnetic bead fresh tissue DNA extraction kit, according to the manufacturer’s instructions. Amplification and sequencing of partial cytochrome oxidase I (COI) and the large nuclear ribosomal subunit (28S) gene sequences were conducted using the primers pairs LCO1490 (5’-GGTCAACAAATCATAAAGATATTGG-3’), HCO2198 (5’-TAAACTT CAGGGTGACCAAAAAATCA-3’), 28S Rd 4.8a (5’-ACCTATTCTCAAACTTTAAATGG-3’), and 28S Rd 7b1 (5’-GACTTCCCTTACCTACAT-3’) described by Folmer et al. (1994) and Robertson et al. (2013), which were synthesized commercially by the General Biology Corporation (Anhui, China). Reads were assembled using DNAMAN 9.0.1 (www.lynnon.com)., and the obtained sequences were used to search the GenBank (www.ncbi.nlm.nih.gov/genbank) and BOLD (boldsystems.org) databases to identify potentially similar species. Sequences of the COI and 28S genes of *P.metasequoiae* sp. nov. have been deposited in the GenBank database to enable molecular diagnosis (COI: PQ276048, 28S: PQ278886).

## ﻿Results

### ﻿Taxonomy

#### ﻿ Phloeosinus metasequoiae

Taxon classificationAnimalia

Ning sp. nov.

7ECC22C0-0D8E-5FE8-B1D1-87E0FE8FB199

https://zoobank.org/16EB30DA-8C67-4444-8719-69B8A1B3EA61

Fig. 1

##### Type material.

***Holotype***: • female: China, Hubei Province: Enshi Tujia and Miao Autonomous Prefecture, Lichuan City, Xindoushan National Nature Reserve, Zhonglu Town, Shiziba Village; 30°7'1"N, 108°41'40"E; elev. 1123 m; all specimens were collected by HN, RD and KX; 5 June 2024; ASM-HBMZU, XDS-SZB007.

***Paratypes***: • 4 females, 5 males, with the same data as the holotype ASM-HBMZU, XDS-SZB003, XDS-SZB011–018 • 3 females, 4 males; Enshi Tujia and Miao Autonomous Prefecture, Lichuan City, Xindoushan National Nature Reserve, Fubao Mountain Forest Management Area; 30°12'33"N, 108°42'26"E; elev. 1394 m; 5 June 2024; ASM-HBMZU, XDS-FBM017–023 • 7 females, 4 males; Enshi Tujia and Miao Autonomous Prefecture, Lichuan City, Xindoushan National Nature Reserve, Wangying Town; 30°16'N, 108°42'17"E; elev. 1130 m; 6 June 2024; ASM-HBMZU, XDS-WY003–008, XDS-WY015–019 • 5 females, 3 males; Enshi Tujia and Miao Autonomous Prefecture, Lichuan City, Xindoushan National Nature Reserve, Jiannan Town; 30°26'9"N, 108°32'15"E; elev. 974 m; 7 June 2024; ASM-HBMZU, XDS-JN010–017 • 7 females, 3 males; Enshi Tujia and Miao Autonomous Prefecture, Lichuan City, Xindoushan National Nature Reserve, Moudao Town; 30°26'N, 108°41'18"E; elev. 1377 m; 8 June 2024; ASM-HBMZU, XDS-MD015–024 • 1 female, 3 males; Enshi Tujia and Miao Autonomous Prefecture, Lichuan City, Xindoushan National Nature Reserve, Zhonglu Town; 30°″2'35"N, 108°44'20"E; elev. 829 m; 9 June 2024; ASM-HBMZU, XDS-ZL002, XDS-ZL008–0010.

##### Similar species.

*Phloeosinusaubei* (Perris, 1855).

##### Diagnosis.

**Figure 1.**
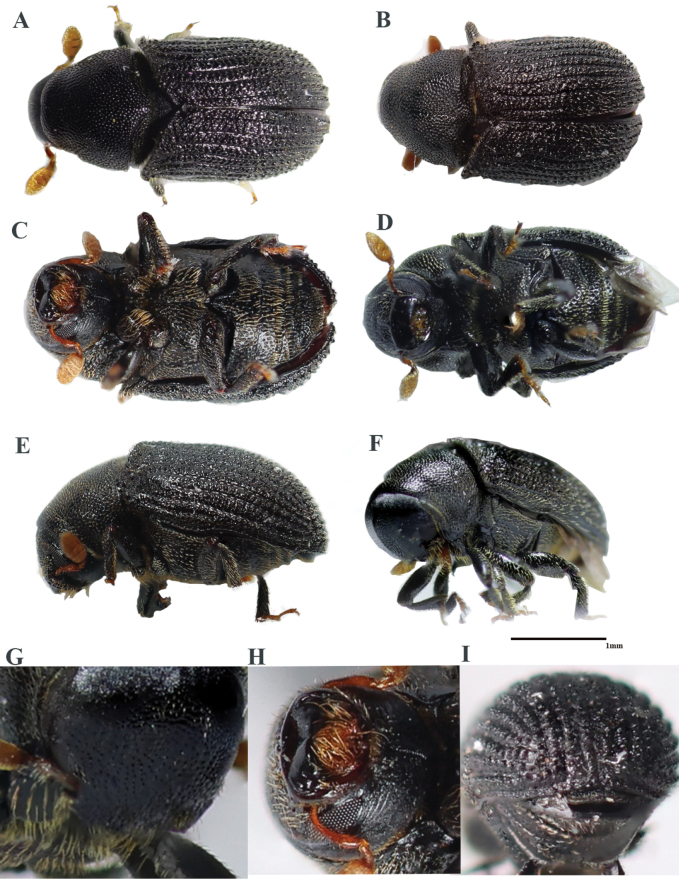
*Phloeosinusmetasequoiae* sp. nov., holotype, female **A, B** dorsal view **C, D** ventral view **E, F** lateral view **G** head **H** mouthparts **I** elytra declivity.

**Figure 2.**
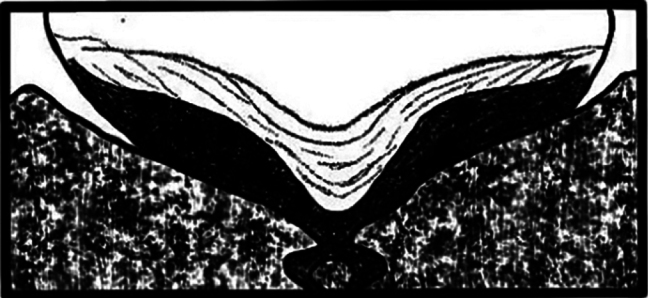
Morphological characteristics of the leading edge of elytra in *Phloeosinusmetasequoiae* sp. nov.

**Figure 3.**
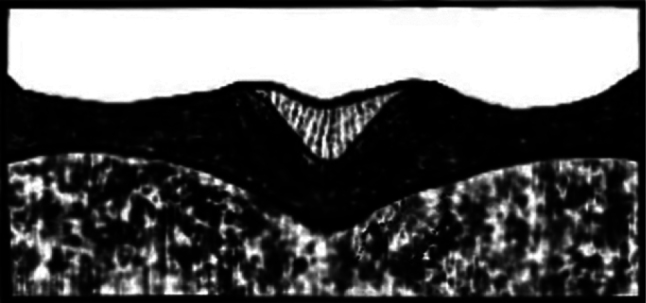
Morphological characteristics of the leading edge of elytra in *Phloeosinusaubei*.

2.2–2.6 mm long (mean = 2.43 mm, *n* = 50); 2.59–2.65× as long as wide. *Phloeosinusmetasequoiae* sp. nov. is similar to *P.aubei*, although the body is shorter and broader, with a notch in the middle of the anterior edge of the compound eye, and an oblong-ovate antennal club; the surface of the anterior thoracic dorsal plate is flat, and there are large tubercles on stripes 1 and 3 of the elytra, as shown in Fig. 1. *Phloeosinusmetasequoiae* sp. nov. can be distinguished from other species of *Phloeosinus* based on two main differences. Firstly, the compound eyes of *P.metasequoiae* sp. nov. are notched, and the frontal and prothoracic surfaces are characterized by reticulations, which is a feature that can be used to distinguish this species from the most similar species, *P.aubei.* Secondly, *P.metasequoiae* sp. nov. and *P.aubei* differ significantly with respect to the basal margin of the elytra, with the basal margin of the *P.metasequoiae* sp. nov. elytra being characterized by its deep V-shape, whereas that of the *P.aubei* elytra is relatively flat, as shown in Figs 2, 3.

##### Description.

**Figure 4.**
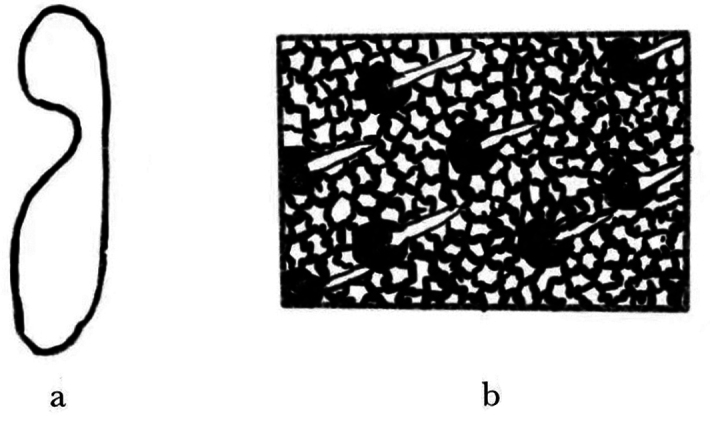
**a** compound eyes deeply emarginate **b** Surface of frons andprothorax not smooth but reticulate.

**Female**: 2.2–2.6 mm long (mean = 2.43 mm, *n* = 50); 2.59–2.65× as long as wide. Body black or black-brown without luster, moderately densely covered with short, yellowish-brown, hair-like setae. ***Head***: epistoma entire, transverse, with a row of moderately long, sparse hair-like setae. Frons below upper margin of eye and above epistoma flat. Eye emargination (Fig. 4). Antennae with four funicle segments (including the pedicel). Antennal club with two procurved sutures. ***Pronotum***: 0.96–1.14 (mean = 1.05, *n* = 50) × as long as wide. Pronotum widest at base, appearing almost triangular in profile. Surface of pronotal plate rough, reticular, with short yellow setae. Apparent triangular protrusion in the pronotum base, curved laterally. ***Elytra***: 1.86–2.04× (mean = 2.0, *n* = 50) × as long as wide, 1.10–1.15 (mean = 1.12, *n* = 50) × as long as pronotum. Deeply V-shaped in the basal margin of elytra. Coarse surface with tubercles, dark-red declivity, and short, yellow setae. Two longitudinal tubercles present between each striae, with nine longitudinal interstriae on each elytra, larger tubercles at the base than at the front end, and larger tubercles in the 1^st^, 3^rd^, and 5^th^ striae near the elytral striae. ***Legs***: procoxae contiguous. Protibiae obliquely triangular, broadest at apical 1/3, densely covered with yellow setae. Posterior face of protibiae, with some small punctures near base and inner margin.

**Figure 5.**
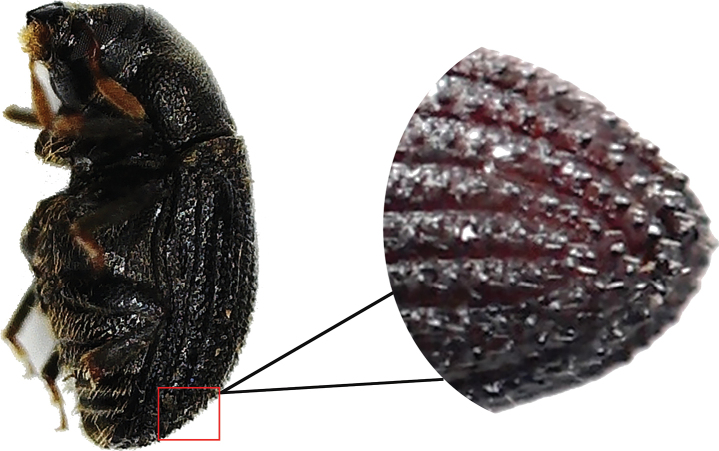
Elytral declivity in *Phloeosinusmetasequoiae* sp. nov. male.

**Figure 6.**
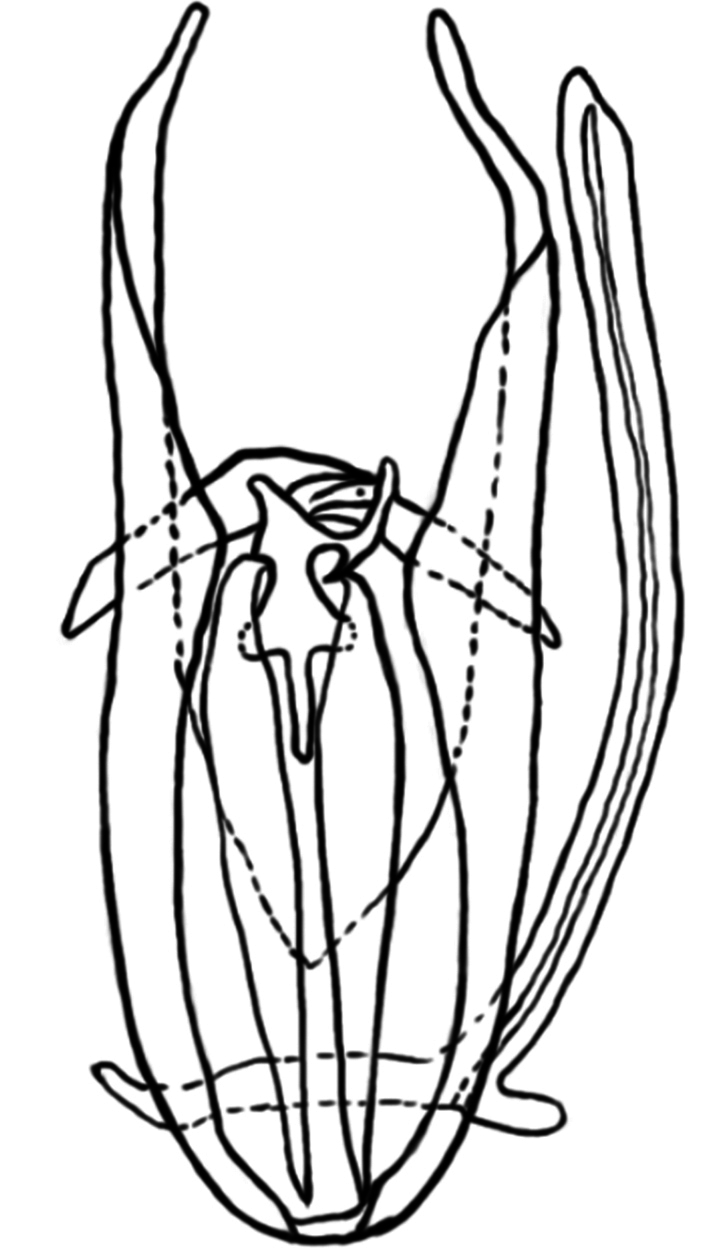
Aedeagus of *Phloeosinusmetasequoiae* sp. nov. male.

**Figure 7.**
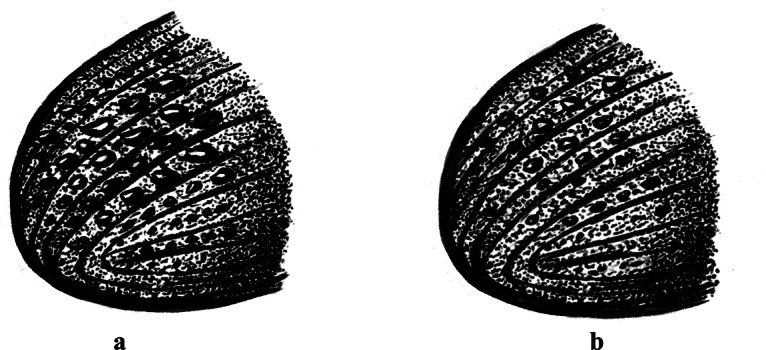
Elytral declivity of *Phloeosinusmetasequoiae* sp. nov. **a** male **b** female.

**Male**. (Figs 5, 6). 2.0–2.5 mm long (mean = 2.38 mm, n = 50); 2.51–2.59× as long as wide. ***Pronotum***: 0.89–1.10 (mean = 0.99, *n* = 50) × as long as wide. ***Elytra***: 1.82–1.97 (mean = 1.91, *n* = 50) × as long as wide, 1.01–1.09 (mean = 1.06, *n* = 50) × as long as pronotum. Coarse surface with tubercles, tubercles in male elytral declivity larger than those in females (Fig. 7). Similar to females in most features except body size and size of tubercles in elytral declivity.

##### Etymology.

The specific epithet *metasequoiae* is the genitive of the genus name of the host plant, *Metasequoia*, indicating that this new species is associated with this plant.

##### Host.

*Metasequoiaglyptostroboides* Hu &W.C.Cheng

##### Distribution.

**Figure 8.**
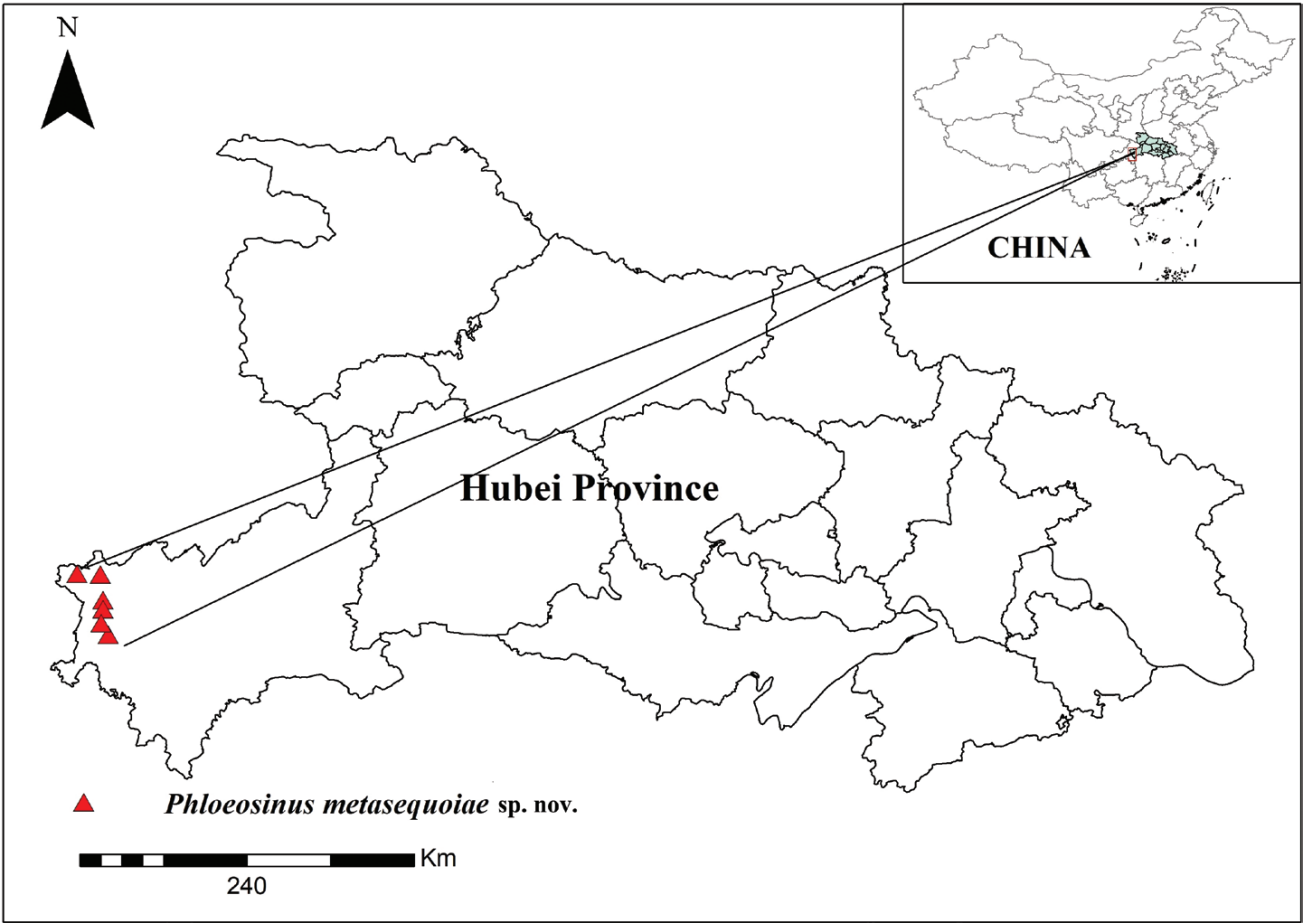
Distribution map of *Phloeosinusmetasequoiae* sp. nov. in China.

Known only from the type locality (Fig. 8).

##### Biology.

Unknown

### ﻿Phylogeny

**Figure 9.**
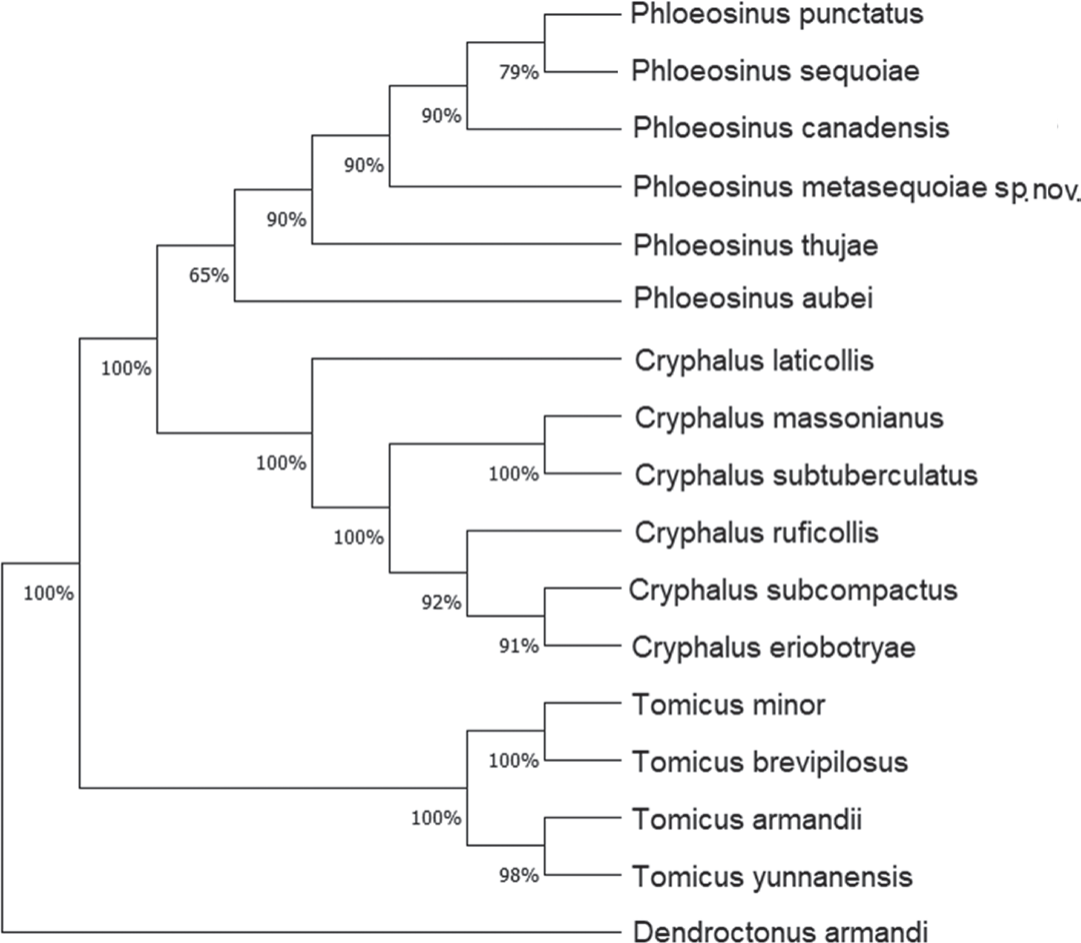
Neighbor-joining tree of analyzed CO1 sequences using the Kimura 2-parameter model, with *Dendroctonusarmandi* as the outgroup.

**Figure 10.**
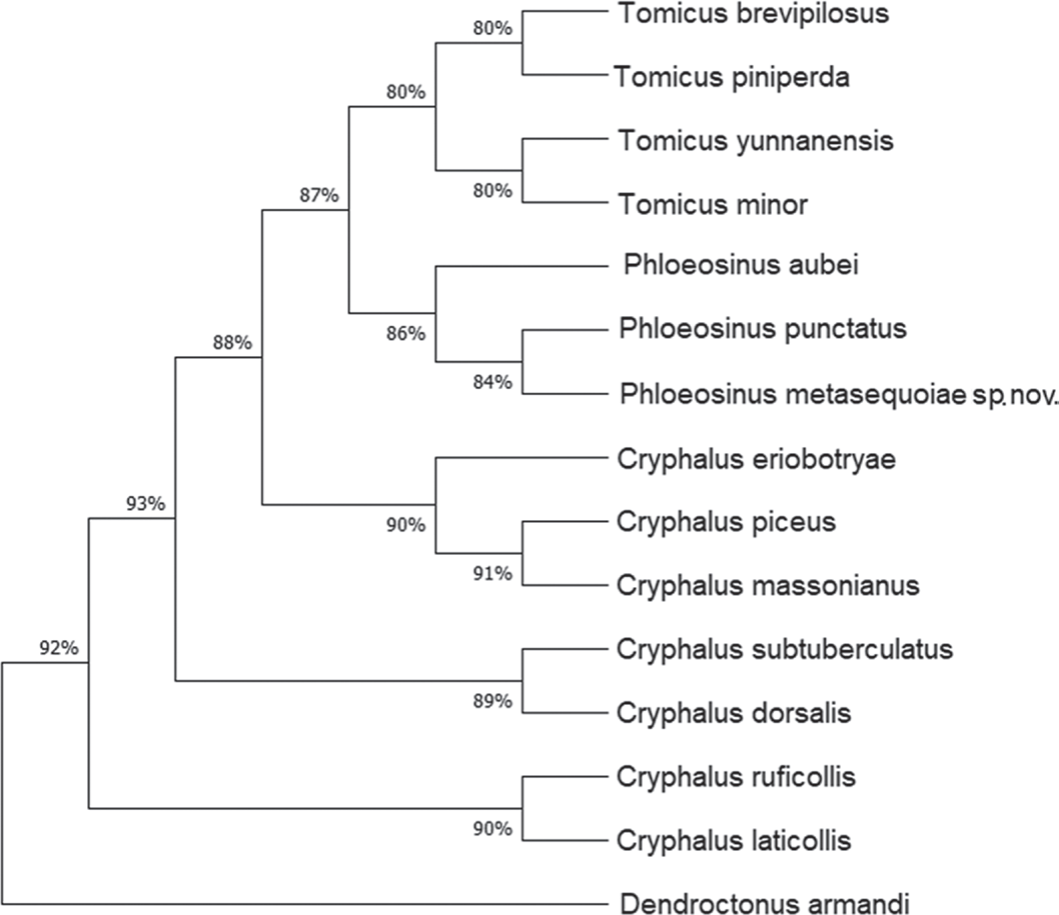
Neighbor-joining tree of analyzed 28S sequences using the Kimura 2-parameter model, with *Dendroctonusarmandi* as the outgroup.

Phylogenetic results (Figs 9, 10) reveal that the closest matching sequences are those of *P.punctatus* (from NCBI, COI: MG054750.1, 86.45% similarity) and *P.aubei* (from NCBI, 28S: JX263746.1, 95.10% similarity). The latter species has a 95% similarly to the *P.metasequoiae* sp. nov., indicating that it is genetically most closely related to *P.aubei*.

## ﻿Discussion

Bark beetles in the genus *Phloeosinus* are widely distributed worldwide. The recently published Cooperative Catalogue of Palaearctic ColeopteraCurculionoidea includes 956 species of Scolytinae in 107 genera and 21 tribes (Knížek 2011; Alonso-Zarazaga et al. 2023). In terms of morphological characteristics, *P.metasequoiae* sp. nov. is most similar to bark beetles within the genus *Phloeosinus*. Distinctive features include a comparatively short, broad body, an emargination in the middle of the anterior edge of the compound eye, and oblong-ovate antennal clubs. The surface of the anterior thoracic dorsal plate is flat, with large tubercles on the first and third interstriae of the elytra. Males and females have concave and convex foreheads, respectively, and genetically, *P.metasequoiae* sp. nov. is most closely related *P.aubei*, with a similarity of 95%.

Having characterized this previously undescribed bark beetle, we immediately conducted an overall survey of *M.glyptostroboides* populations in the vicinity of Lichuan City, and to date, bark beetles have been found in the Xiaohe *M.glyptostroboides* Botanical Gardens, Zhonglu Town, and Jiannan Town. In Shiziba Village, near the Xiaohe *M.glyptostroboides* Botanical Gardens, our investigations revealed that at least seven mother trees were infected with the new species.

**Figure 11.**
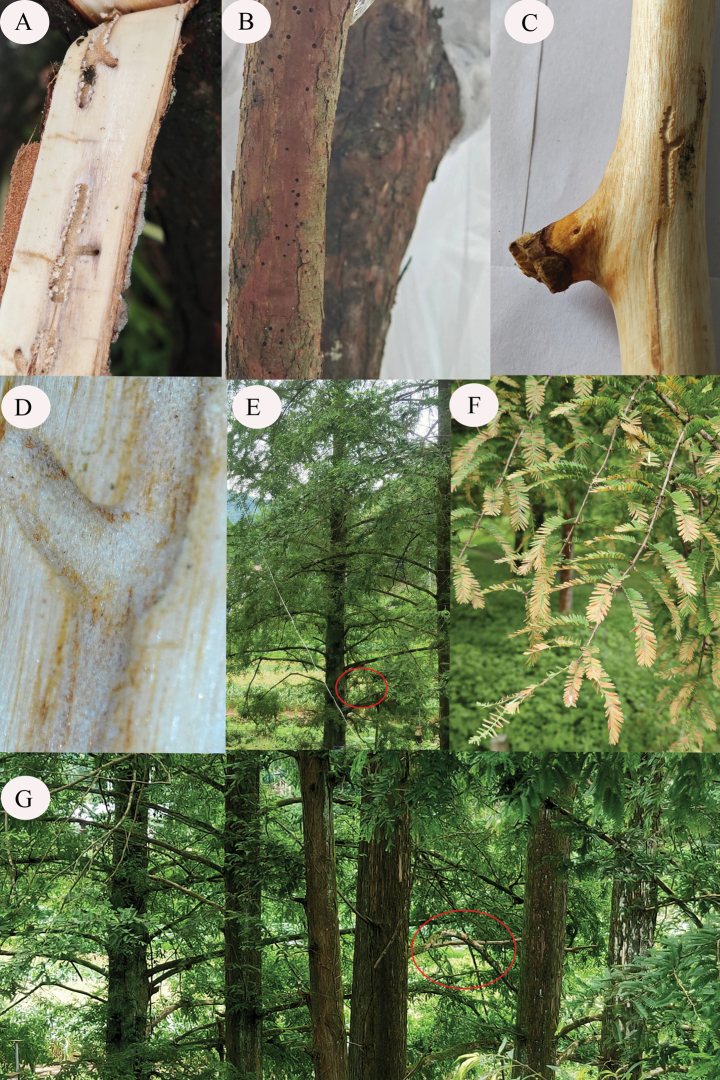
**A** galleries on the underside of bark **B** invasion holes in tree branches **C, D***Phloeosinusmetasequoiae* sp. nov. galleries in tree branches **E–G** trees infested with *P.metasequoiae* sp. nov.

Our survey reveals that the initial invasion of *M.glyptostroboides* by *P.metasequoiae* sp. nov. can be detected by the appearance of small circular holes measuring less than 0.5 mm in the branches of host trees (Fig. 11B), and that as infestation progresses, the branches begin to show signs of decay, as evidenced by the gradual deterioration shown in Fig. 11E, G. Tunnels excavated by the adult beetles are longitudinal pits that extend primarily into the sapwood and, in most cases, terminate in a short horizontal pit. We established that the length of these tunnels tends to be proportional to the density of the invading bark beetles, typically ranging from 30 to 50 mm, with minimum and maximum lengths of 20 and 95 mm, respectively, and a width of approximately 1–2 mm (Fig. 11A, C, D). The most apparent symptom of the damage caused by this bark beetle is a reddening of the leaves during summer, which can be readily identified and is conducive to sampling by cutting branches (Fig. 11F). However, during the initial stages of infestation, symptoms are less apparent, and we thus speculate that there may have been more infected mother trees than those identified in our survey. Among cultivated *M.glyptostroboides*, we similarly detected the presence of this bark beetle in up to 20 infected trees, which, if we consider the occurrence of asymptomatic initially infected trees, could represent a minimum number. In addition, given the sporadic distribution of *M.glyptostroboides*, it is plausible that some trees may have been overlooked and, consequently, the actual number of infected trees may be considerably less significant.

We also conducted a comprehensive investigation of potential host trees, focusing primarily on species within the families Cupressaceae and Taxodiaceae. However, we failed to detect this beetle in any tree species other than *M.glyptostroboides*, thereby indicating that it may be an obligate pest of this tree. A further investigation assessing the environments surrounding infected trees reveals that damage was most severe among those trees growing along national roads, which were characterized by relatively poor vigor compared with *M.glyptostroboides* trees distributed in localities far from these roads. In this regard, we note that *M.glyptostroboides* growing along national roads tend to be regularly pruned, as excessively long branches can obstruct the view of vehicles, thereby influencing the growth of these trees, and we found that bark beetles preferentially invaded healthy trees with weaker vigor and diameter at breast height ranging from 50 to 80 cm (Fig. 11E–G).

## Supplementary Material

XML Treatment for Phloeosinus metasequoiae

## ﻿Additional information

### Data availability

All of the data that support the findings of this study are available in the main text.
